# Maternal common mental disorders and infant development in Ethiopia: the P-MaMiE Birth Cohort

**DOI:** 10.1186/1471-2458-10-693

**Published:** 2010-11-12

**Authors:** Chiara Servili, Girmay Medhin, Charlotte Hanlon, Mark Tomlinson, Bogale Worku, Yonas Baheretibeb, Michael Dewey, Atalay Alem, Martin Prince

**Affiliations:** 1 Department of Psychiatry, University of Modena and Reggio Emilia, Italy; 2 Aklilu Lemma Institute of Pathobiology, Addis Ababa University, Addis Ababa, Ethiopia; 3 Department of Psychiatry, Faculty of Medicine, Addis Ababa University, Addis Ababa, Ethiopia; 4 King's College London (Institute of Psychiatry), Health Service and Population Research Department, De Crespigny Park, London, UK; 5 Department of Psychology, Stellenbosch University, Matieland, South Africa; 6 Department of Paediatrics and Child Health, Addis Ababa University, Addis Ababa, Ethiopia

## Abstract

**Background:**

Chronicity and severity of early exposure to maternal common mental disorders (CMD) has been associated with poorer infant development in high-income countries. In low- and middle-income countries (LAMICs), perinatal CMD is inconsistently associated with infant development, but the impact of severity and persistence has not been examined.

**Methods:**

A nested population-based cohort of 258 pregnant women was identified from the Perinatal Maternal Mental Disorder in Ethiopia (*P-MaMiE*) study, and 194 (75.2%) were successfully followed up until the infants were 12 months of age. Maternal CMD was measured in pregnancy and at two and 12 months postnatal using the WHO Self-Reporting Questionnaire, validated for use in this setting. Infant outcomes were evaluated using the Bayley Scales of Infant Development.

**Results:**

Antenatal maternal CMD symptoms were associated with poorer infant motor development ($\hat{\beta }$ -0.20; 95% CI: -0.37 to -0.03), but this became non-significant after adjusting for confounders. Postnatal CMD symptoms were not associated with any domain of infant development. There was evidence of a dose-response relationship between the number of time-points at which the mother had high levels of CMD symptoms (SRQ ≥ 6) and impaired infant motor development ($\hat{\beta }$ = -0.80; 95%CI -2.24, 0.65 for ante- or postnatal CMD only, $\hat{\beta }$ = -4.19; 95%CI -8.60, 0.21 for ante- and postnatal CMD, compared to no CMD; test-for-trend χ^2^13.08(1), p < 0.001). Although this association became non-significant in the fully adjusted model, the $\hat{\beta }$ coefficients were unchanged indicating that the relationship was not confounded. In multivariable analyses, lower socio-economic status and lower infant weight-for-age were associated with significantly lower scores on both motor and cognitive developmental scales. Maternal experience of physical violence was significantly associated with impaired cognitive development.

**Conclusions:**

The study supports the hypothesis that it is the accumulation of risk exposures across time rather than early exposure to maternal CMD per se that is more likely to affect child development. Further investigation of the impact of chronicity of maternal CMD upon child development in LAMICs is indicated. In the Ethiopian setting, poverty, interpersonal violence and infant undernutrition should be targets for interventions to reduce the loss of child developmental potential.

## Background

There is evidence indicating that maternal common mental disorders (CMD), in particular depressive and anxiety disorders, pose a serious public health concern because of their adverse effect on infant development [1,2]. Infants of mothers with CMD have been found to have poorer motor, cognitive and socio-emotional development than children of mothers in good mental health in many [3,4], but not all [5], studies. In an attempt to explain these heterogeneous findings, the influence of chronicity, severity and timing of maternal CMD [2,5-7] and the role of mediating factors upon child developmental outcomes have been explored [8-10]. The postnatal period may be a critical time for exposure to maternal CMD, leading to enduring effects on subsequent child development [11-13]. Evidence is also emerging of a negative impact of antenatal CMD symptoms upon infant development [3]. However, a recent review [14] argued that the children most at risk of developmental deficits are those of mothers exhibiting depressed mood throughout their early childhood years. The pattern of less sensitive and responsive parenting, characteristic of chronically depressed mothers, may represent a point of entry onto developmental pathways of risk, especially for cognitive and language problems [7]. Socio-economic factors and infant male sex appear to exacerbate the effect of maternal CMD [8,12,15-18].

Findings from the few studies to have explored the impact of postnatal CMD upon infant development in LAMIC settings are largely congruent with evidence from high-income countries. In hospital-based, urban cohort studies of six month old infants from India [19] and Barbados [20], as well as a rural, cross-sectional population-based study of three to 24 month old children in Ethiopia [21], postnatal CMD was independently associated with poorer infant developmental outcomes (particularly cognitive) after adjusting for confounders. However, a prospective, rural, population-based study from Bangladesh only found an association when the mother also perceived her infant's temperament to be difficult [22]. These studies have been limited by the use of non-validated measures of maternal CMD [20-22]. None have evaluated the impact of maternal antenatal CMD or the effect of chronicity of maternal CMD upon infant development. Furthermore, not all studies made adequate adjustment for potential confounders [19].

In this paper, we present findings from a large, population-based cohort study from rural Ethiopia; the *P*erinatal *Ma*ternal *M*ental Disorder *i*n *E*thiopia (*P-MaMiE*) study. The aims of the analyses presented in this paper were as follows:

1. To investigate the effect of maternal CMD on child developmental outcomes. Two main hypotheses were tested. First, that the association between maternal postnatal CMD and impaired infant cognitive development found in other LAMICs would pertain in our setting. Second, that antenatal CMD would be associated with poorer infant development, independently of postnatal CMD. Our dataset also allowed us to explore the effect of chronicity and timing of exposure to maternal CMD in the first year upon developmental outcomes at 12 months of age.

2. Explore the role of other potential risk factors for child development.

## Methods

A population-based sample of 1065 women in the third trimester of pregnancy was recruited between July 2005 and March 2006. The women were followed up until their infants were 12 months of age.

### Setting

The *P-MaMiE *study was situated in the demographic surveillance site (DSS) at Butajira, a predominantly rural area of Ethiopia located 130 km south of the capital city Addis Ababa. The DSS is part of the Butajira Rural Health Programme (BRHP) http://www.indepth-network.org/dss_site_profiles/butajira.pdf .

### Sample

**Figure 1:**
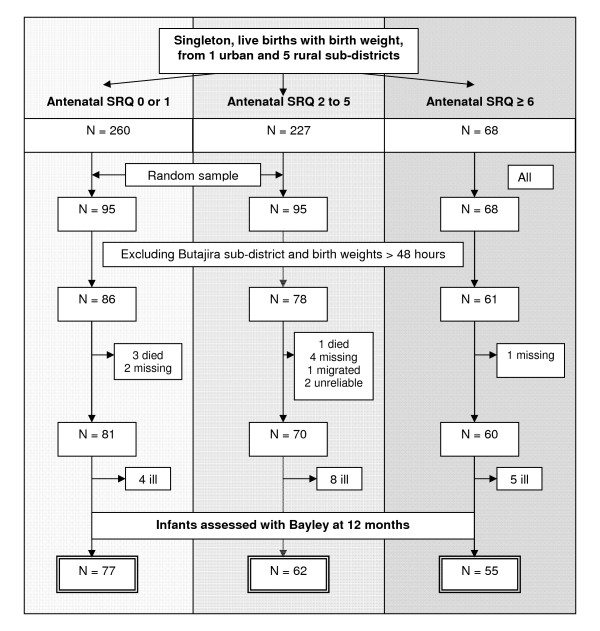
**Flow diagram showing sampling of infants for Bayley scale assessment**.

The *P-MaMiE *study sample size was determined to address objectives other than those addressed within this paper. For the study presented in this paper, the sampling frame was restricted to infants who were weighed at birth (n = 555). Birth weight was only measured in sub-districts where a suitable health worker could be identified, as previously reported [23]. However, due to low birth weight coverage in the urban sub-district, infants from this sub-district were subsequently excluded from the study. Babies not weighed within 48 hours of birth were also excluded due to the unreliability of birth weight measurements in this group. Overall, birth weight coverage was 79.7% of the remaining eligible babies (n = 521). Women whose infant had birth weight measured within 48 hours of birth did not differ from those whose baby was measured after 48 hours in terms of levels of maternal CMD or on a number of other important variables, as previously reported [23]. A sub-sample of infants who had been weighed within 48 hours of birth was selected for developmental assessment, stratified by level of exposure to maternal antenatal CMD symptoms (see figure 1). All infants exposed to high levels of antenatal CMD symptoms (≥6 on the Self-Reporting Questionnaire-20 (SRQ) [24]) (n = 68) and a random sub-sample of infants exposed to moderate (SRQ score 2 to 5; n = 95/227) and low (SRQ score 0 or 1; n = 95/260) antenatal CMD symptoms were selected for assessment. The number of infants included in the comparison groups was limited by feasibility considerations.

### Measures

#### Infant development

Three domains of infant development were measured using the Bayley Scales of Infant Development, third edition ('the Bayley scales'): cognitive, language and motor [25]. The earlier version of the Bayley scales (version II) has been used in two previous published studies from Ethiopia, although has never been formally validated in this setting [26,27].

#### Adapting the Bayley Scales

Considerable time was invested into ensuring a good translation of the items into Amharic, the official language of Ethiopia, and pilot testing the Bayley Scales on one year old infants from the Butajira area. Three items from the motor sub-scale were dropped as they involved use of stairs. The participants' homes are single-storey dwellings without steps and are located several kilometres from the road, making it impractical to carry pre-made stairs to each house. Following the example of a previous Ethiopian study [26], we did not impose limits for completion of timed items.

#### Training of Bayley Scale administrators

The Bayley Scales were administered by nurses (n = 5) and *P-MaMiE *data collectors (n = 2, high school completers). It has previously been demonstrated that high school graduates can be successfully trained to administer the Bayley Scales in Ethiopia [27]. The Bayley Scale administrators received intensive training over ten days from the project co-ordinator and an Ethiopian Consultant Paediatrician (BW). Training included simultaneous ratings of Bayley administrations and detailed discussion of any discrepancies. At the end of training, seven out of eight participants were considered competent. After commencement of data collection, regular field checks were carried out to ensure continued quality of administration.

#### Bayley administration

All Bayley scale assessments were carried out in the mother's home. The infant's mother was present in all cases and was free to breastfeed throughout. Bayley administrators were unaware of the mother's mental health status.

#### Maternal mental health

Maternal CMD was measured antenatally and at two and twelve months post-natally using the SRQ-20 [24]. This 20-item scale asks about depressive, anxiety and somatic symptoms present in the preceding month, generating a continuously distributed scale score indicating level of overall psychological morbidity. The SRQ was extensively pre-validated for use in a mixed sample of pregnant and postnatal women in the Butajira population [28].

#### Potential confounders, mediators and effect modifiers

**Figure 2:**
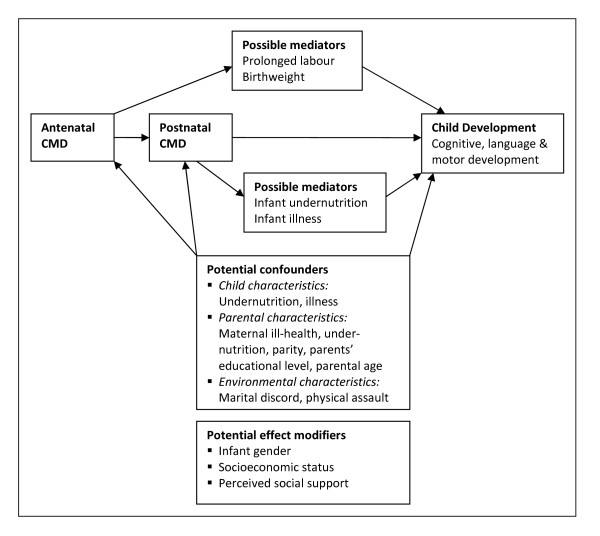
**Conceptual framework for the association between perinatal CMD and child development**.

A conceptual model for the relationship between antenatal/postnatal CMD and child development is illustrated in Figure 2.

Potential confounders were identified from the literature (referenced) as well as possible confounders identified on a theoretical basis (marked with *).

(a) Background: Socio-economic status [8,21] was measured by asking about (i) hunger in the preceding month due to lack of resources, and (ii) subjective report of wealth relative to others. Parity [29], parental age [21] and educational level [26,27] were obtained through self-report. Marital discord [11] was indicated through self-report that (i) the husband not providing enough help, (ii) the relationship was 'average, bad or very bad', (iii) they sometimes or often quarrelled, and (iv) the woman felt that her husband drank too much alcohol. A hierarchical non-parametric marital discord scale was confirmed using Mokken analysis [30] of these four items (Loevinger H coefficient 0.35), as previously described [31]. (b) Pregnancy time-point: Self-report data was obtained on physical assault during pregnancy [1] and episodes of malaria [32]. Poorer perceived social support was indicated through self-report that (i) not attending groups, (ii) less frequent visit to friends,(iii) not enough help in house, (iv) not enough help to look after children*. Maternal weight and height were measured in pregnancy using standard anthropometric techniques [33] and used to calculate maternal body mass index* (kg/m^2^).

(b) Pregnancy time-point: Self-report data was obtained on physical assault during pregnancy.

(c) Birth time-point: Infant gender [12,34].

(d) Two months postnatal time-point: Maternal ill-health* was indicated by self-report of episodes of diarrhoea or malaria experienced since birth. Poorer social support* was measured as in pregnancy.

(e) Twelve months postnatal: Maternal CMD was measured using the SRQ-20.

Potential mediators:

(a) Birth weight [34] was measured using SECA 725 scales measuring to an accuracy of 10 g.

(b) Maternal report of prolonged labour (longer than 24 hours) was obtained at the birth time-point.

(c) Infant nutritional status: Infant weight was measured using SECA725 scales as for birth weight. Length was measured using a locally-constructed infantometer, accurate to 0.1 cm. In the analyses we used weight-for-age rather than height-for-age z-scores, as this as been argued to be a more useful summary indicator [35].

(d) Maternal recall of number of infant diarrhoeal episodes since birth [36,37].

Potential effect modifiers:

Socioeconomic status and child gender were considered to be potential effect modifiers on the basis of previous studies [12,15,16,18]. Social support was conceptualised as a potential effect modifier on theoretical grounds. However, all three variables could also be operating as confounding variables. We therefore included these variables as potential confounders in the initial analyses, but conducted further exploratory analyses to look for evidence of effect modification.

### Ethical considerations

Ethical approval was obtained from the National Ethical Review Committee for Ethiopia and the Research Ethics Committee of King's College London in the UK. Written, informed consent was obtained. All women were reimbursed for health care costs for themselves and their infant. Those women who were suffering from severe mental disturbance were referred for assessment at the local psychiatric unit in Butajira town.

### Data analysis

For all analyses, the data were weighted back according to the reciprocal of the achieved sampling fraction (68/55 for high antenatal CMD exposure, 227/62 for moderate CMD exposure and 260/77 for low CMD exposure) using Stata version 10 [38]. Robust standard errors were calculated to account for weighting. Raw Bayley scores were used in all analyses. The initial analysis strategy was hypothesis-driven, using linear regression to investigate the association between exposures of (1) postnatal maternal CMD and (2) antenatal maternal CMD upon infant developmental outcomes. Maternal CMD was added into the model as a continuous exposure variable (SRQ score) in order to maximise power to detect an effect. For the model including antenatal CMD as primary exposure, birth weight and prolonged labour were conceptualised as potentially lying on a causal pathway from antenatal CMD to child development. Infant undernutrition and illness episodes were considered to be probable mediators of any effect of postnatal CMD on child development. Therefore, these variables were not included as potential confounders but were individually added to the final multivariable model to look for preliminary evidence of a mediating role. The potential role of effect modification was explored by including interaction terms in three separate analyses: maternal CMD x socio-economic status, maternal CMD x infant gender, and maternal CMD x perceived social support.

To explore the possible impact of persistent (chronic) maternal CMD, two composite variables were created using the SRQ score cut-off of ≥6 to indicate high levels of CMD symptoms, as supported by our validation study [28]. 'Persistent perinatal CMD' was defined as high CMD in pregnancy and at 2 months postnatal. 'Persistent maternal CMD' was defined as CMD at 2 and 12 months postnatal. Multivariable linear regression analyses were then conducted as above.

Subsequently, an exploratory analysis was conducted to identify the range of variables associated with infant development in this setting. All potential predictors of infant development were included in the final fully adjusted model regardless of whether they were associated in bivariate analyses.

## Results

A flow chart of infants followed up to 12 months of age who underwent developmental assessment with the Bayley Scales is presented in Figure 1. Of those infants with birthweight measured within 48 hours of birth who had been selected for assessment, 86.2% (194/225) are included in the following analyses. The reasons for non-inclusion are as follows: four infants died, seven were not traced, one mother out-migrated, two had Bayley assessments that were considered unreliable, and 17 were observed to be ill at the time of testing.

Developmental assessments were completed for 81.1% of infants in the low antenatal CMD exposure group (maternal SRQ score < 2), 65.3% in the moderate CMD exposure group (SRQ score 2 to 5) and 80.9% in the high CMD exposure group (SRQ score ≥ 6). There was significantly higher differential loss from the category of moderate CMD exposure compared to both high (χ^2^(df 1) = 4.77, p = 0.003) and low (χ^2^(df 1) = 6.03, p = 0.014) CMD exposure. However, there was no significant difference in baseline characteristics in those lost to follow-up. Every infant was assessed at the age of 12 months +/- one week. The median duration of one Bayley scale assessment was 1 hour and 10 minutes, ranging from 20 minutes up to 2 hours and 30 minutes.

### Sample characteristics

Table 1 shows the background characteristics of participating women and their infants.

**Table 1:** Characteristics of participating mothers and infants (n = 194)

Characteristics	N (%)
Maternal SRQ score: Median (25^th^, 75^th^) Pregnancy	2(1,6)
2 months postnatal	1(0,3)
12 months postnatal	1 (0,3)
Maternal age (years). Mean (SD)	27.4 (6.39)
Husband's age (years). Mean (SD)	36.8 (9.33)
No maternal education	170 (87.6)
No paternal education	64 (33.2)
Parity: Primiparous	24 (12.4)
2 to 4 previous live births	77 (39.7)
5 or more previous live births	93 (47.9)
Lower relative wealth of the household	111 (57.2)
Hungry in the last 30 days	38 (19.6)
Marital discord scale (0 to 4). Median (25^th^, 75^th^)	0 (0,1)
Physically assaulted	7 (3.6)
One or more malarial episodes in pregnancy	37 (19.2)
Maternal BMI (kg/m^2^) Mean (SD): Pregnancy	21.6 (2.37)
Postnatal 12 months	20.8 (2.9)
Sees friends ≤ monthly: Pregnancy	39 (20.1)
Postnatal 2 months	32 (16.7)
Birth weight (kg) Mean (SD)	3.0 (0.4)
Prolonged labour	36 (18.6)
Episodes of fever since birth	57 (29.7)
Episode of diarrhoea since birth	14 (7.3)
Infant gender female	94(48.5)
Infant diarrhoeal episodes in 1^st ^year: 2 or more	104 (53.6)
Weight-for-age Z score in infant. Mean (SD)	-1.02 (1.35)

SRQ = Self-reporting questionnaire, 20-item version; BMI = Body Mass Index

At 12 months of age, the prevalence of infants who were underweight (<-2 for Weight-for-Age Z-score) was 41 (21.6%) and 86 (45.5%) were stunted (<-2 for Height-for-Age Z-score).

Only 14 (7.3%) of women had persistent perinatal CMD (SRQ ≥ 6 in pregnancy and at 2 months postnatal), 42 (21.9%) had CMD at either of the perinatal time-points and 139 (70.8%) did not have CMD at either perinatal time-point. In the first postnatal year, 8 (4.1%) of women had persistent maternal CMD (present at both 2 and 12 months postnatal), 18 (9.3%) had CMD at only one postnatal time-point, and 168 (86.6%) did not have CMD at either postnatal time-point.

The mean scores (SD) for the sub-scales of the Bayley were as follows: cognitive 19.2 (3.30), language 20.4 (3.65) and motor 35.6 (4.88).

### Maternal CMD symptoms and infant development

In both univariate and multivariable linear regression analyses, no association was found between maternal postnatal CMD symptoms (SRQ score) and any of the Bayley developmental sub-scales. When antenatal CMD symptom score was modelled as the primary exposure, an association was seen with motor developmental scores in the univariate analysis ($\hat{\beta }$ -0.20; 95% CI: -0.37 to -0.03), but this became non-significant in the final adjusted model (Table 2).

**Table 2:** Antenatal or postnatal CMD and infant development

Characteristics	Cognitive score		Language score		Motor score	
	$\hat{\beta }$	95% CI	$\hat{\beta }$	95% CI	$\hat{\beta }$	95% CI
**Postnatal CMD as primary exposure**						

Unadjusted $\hat{\beta }$	0.06	-0.08,	0.09	-0.09,	-0.23	-0.52,
		0.20		0.27		0.06
Fully adjusted model* (n = 183)	**0.04**	**-0.10**,	**0.001**	**-0.22**,	**-0.23**	**-0.52**,
		**0.17**		**0.22**		**0.06**
Final model adjusted for potential mediators						
						
Infant nutritional status	0.02	-0.12,	-0.004	-0.22,	-0.25	-0.56,
		0.17		0.23		0.05
Infant illness episodes	0.04	-0.10,	-0.001	-0.22,	-0.23	-0.52,
		0.17		0.22		0.06
						

**Antenatal CMD as primary exposure**						

Unadjusted $\hat{\beta }$	-0.08	-0.20,	-0.001	-0.14,	-0.20	-0.37, -
		0.03		0.13		0.03
Final adjusted model* (n = 185)	**-0.05**	**-0.20**,	**-0.01**	**-0.16**,	**-0.01**	**-0.21**,
		**0.09**		**0.17**		**0.19**
Final model adjusted for potential mediators:						
						
Prolonged labour	-0.06	-0.21,	-0.002	-0.16,	0.03	-0.23,
(n = 185)		0.09		0.17		0.17
Birth weight (n = 185)	-0.05	-0.20.	-0.01	-0.16,	-0.01	-0.21
		0.09		0.17		0.19

Association between maternal antenatal/postnatal common mental disorder symptoms (CMD: SRQ score) and scores on Bayley sub-scales for 12 month old infants (n = 194)

*Potential confounders as described in methods

The association between antenatal CMD symptoms and Bayley motor score appeared to be predominantly confounded by parental characteristics, socio-economic status and marital discord. There was no association between antenatal CMD symptoms and either cognitive or language development. There was no evidence that any effect of antenatal CMD upon developmental outcomes was mediated through infant birth weight or maternal experience of prolonged labour.

None of the interaction terms for maternal CMD x socioeconomic status, CMD x infant gender or CMD x social support were significant (results not shown).

### Persistent maternal CMD and infant development

A dose-response relationship was observed between the number of time-points at which the infant was exposed to maternal CMD and scores on the motor developmental scale. For the perinatal period, women with CMD either in pregnancy or at postnatal 2 months had infants with a lower mean motor score ($\hat{\beta }$ -0.80; 95%CI -2.24, 0.65), and the mean motor score was even lower for women with CMD at both time-points ($\hat{\beta }$ -4.19; 95%CI -8.60, 021); test-for-trend χ^2^13.08(1), p < 0.001. Similarly, for infants whose mothers had CMD at both 2 and 12 months postnatal (See Table 3). These associations became non-significant in the final adjusted models, although there was little change in the size of the $\hat{\beta }$ estimates and the confidence intervals were wide. See Table 3.

**Table 3:** Persistent perinatal CMD and infant development

	Persistent perinatal CMD		**Categories of increasing exposure to perinatal CMD**^**¥**^
	$\hat{\beta }$**(95% Confidence Interval)**		
Bayley sub-scale	SRQ ≥ 6 in pregnancy or 2 **months postnatal**^**§**^	SRQ ≥ 6 in pregnancy & 2 **months postnatal**^**§**^	**Test-for-trend Χ**^**2 **^**(df); p-value**
*Cognitive*			
Crude	-0.99 (-2.12, 0.15)	-1.59 (-3.99, 0.82)	6.66(1); p < 0.025
Fully adjusted*	-0.64 (-1.79, 0.51)	-2.11 (-4.87, 0.65)	2.58(1); p > 0.10
*Motor*			
Crude	-0.80 (-2.24, 0.65)	-4.19 (-8.60, 0.21)	13.08(1); p < 0.001
Fully adjusted*	0.15 (-1.52, 1.82)	-4.15 (-9.19, 0.89)	2.83(1); p > 0.05
*Language*			
Crude	-0.16 (-1.45, 1.13)	-0.72 (-3.29, 1.85)	3.93(1); p < 0.05
Fully adjusted*	0.21 (-1.11, 1.53)	-1.12 (-4.42, 2.17)	0.23(1); p > 0.50

	**Persistent postnatal CMD**		**Categories of increasing exposure to postnatal CMD**^¶^
	$\hat{\beta }$ (95% Confidence Interval)		
**Bayley sub-scale**	SRQ ≥ 6 at postnatal 2 OR 12 months^±^	SRQ ≥ 6 at postnatal 2 & 12 months^±^	Test-for-trend Χ^2 ^(df); p-value

*Cognitive*			
Crude	0.61 (-1.28, 2.51)	-0.25 (-1.49, 0.99)	0.04(1); p > 0.50
Fully adjusted*	0.90 (-1.13, 2.93)	0.20 (-1.28, 1.67)	0.29(1); p > 0.50
*Motor*			
Crude	-2.22 (-5.96, 1.52)	-3.01 (-8.01, 2.00)	3.37(1); p > 0.10
Fully adjusted*	-1.42 (-4.91, 2.07)	-2.65 (-9.26, 3.96)	1.75(1); p > 0.10
*Language*			
Crude	2.16 (0.35, 3.96)	-0.61 (-3.66, 2.44)	0.60(1); p > 0.25
Fully adjusted*	2.37 (0.18, 4.55)	-0.56 (-4.01, 2.90)	0.74(1); p > 0.25

Exploratory analyses showing association between persistent maternal common mental disorder (CMD) and infant Bayley scale scores at 12 months of age (n = 183 in adjusted model)

^§ ^Reference category = SRQ < 6 in pregnancy and 2 months postnatal. ^± ^Reference category = SRQ < 6 at 2 and 12 months postnatal. *Adjusted for all potential confounders. ^¥^No CMD, CMD in pregnancy or postnatal (2 months), CMD in both pregnancy and postnatal. ^¶^No CMD, CMD in pregnancy or postnatal (12 months), CMD in both pregnancy and postnatal.

### Multivariable analysis of predictors of infant development

In the multivariable analyses, poorer infant performance on the cognitive sub-scale was associated with lower self-ranking of wealth relative to others ($\hat{\beta }$ -1.50; 95%CI -2.54, -0.45) and maternal experience of physical assault ($\hat{\beta }$ -2.72; 95%CI -4.35, -1.09). Higher infant weight-for-height was associated with better cognitive performance ($\hat{\beta }$ 0.50; 95%CI 0.03, 0.98). See Table 4.

**Table 4:** Multivariable model of predictors of Bayley cognitive scores of 12 month old infants

	***Crude ***$\hat{\beta }$***(95%CI)*** *(n = 194)*	***Fully adjusted***$\hat{\beta }$***(95%CI) Model (n = 183)***
Maternal age (years)	-0.06 (-0.13, 0.01)	-0.07 (0.24, 0.09)
Age of husband (years)	-0.01 (-0.05, 0.04)	0.04 (-0.02, 0.10)
No maternal education	-0.08 (-1.38, 1.22)	-1.34 (-2.82, 0.13)
No paternal education	0.40 (-0.57, 1.36)	0.67 (-0.40, 1.74)
Parity: Primiparous	Ref.^§^	Ref.^§^
2 to 4 births	-0.08 (-1.68, 1.52)	0.08 (-1.61, 1.77)
5 or more	-0.45 (-2.01, 1.12)	0.02 (-2.32, 2.36)
Lower relative wealth	-1.46 (-2.40,-0.53)	-1.50 (-2.54, -0.45)
Hungry in last 30 days	-0.77 (-2.03, 0.49)	-0.23 (-1.80, 1.35)
Marital discord scale	-0.27 (-0.82, 0.28)	-0.04 (-0.67, 0.60)
Physically assaulted	-2.21 (-3.95, -0.47)	-2.72 (-4.35, -1.09)
Female infant gender	-0.65 (-1.60, 0.30)	-1.00 (-2.17, 0.17)
Birth weight (kg)	-0.30 (-1.36, 0.77)	-0.36 (-1.59, 0.87)
***Pregnancy variables***		
Maternal SRQ score	-0.08 (-0.20, 0.03)	-0.07 (-0.21, 0.08)
≥1 malarial episode	-0.65 (-2.01, 0.71)	-0.03 (-1.44, 1.38)
***Postnatal 2 month variables***		
Maternal SRQ score	0.06 (-0.08, 0.20)	0.06 (-0.09, 0.20)
Prolonged labour (>24 hours)	-0.83 (-0.19, 0.27)	-1.03 (-2.22, 0.15)
***Postnatal 12 month variables***		
Maternal SRQ score	0.04 (-0.13, 0.21)	0.07 (-0.16, 0.30)
Infant diarrhoea: 0	Ref.^±^	Ref.^±^
1 episode	0.14 (-1.38, 1.67)	0.58 (-1.31, 2.46)
≥2 episodes	0.13 (-1.12, 1.38)	0.58 (-1.05, 2.22)
Weight-for-Age Z-score	0.35 (-0.01, 0.71)	0.50 (0.03, 0.98)

^§^Test-for-trend for parity: Χ^2 ^0.62(1), p > 0.25 for crude and Χ^2 ^0.0000006(1), p = > 0.50 for adjusted model. ^±^Test-for-trend for infant diarrhoeal episodes: Χ^2 ^0.02(1), p > 0.50 for crude and Χ^2 ^0.41(1), p > 0.50 for adjusted model.

For the motor sub-scale, poorer performance was associated with lower perceived relative wealth in the multivariable model ($\hat{\beta }$ -2.15; 95%CI -3.53, -0.77). Higher infant weight-for-age was significantly associated with better motor performance ($\hat{\beta }$ 0.70; 95%CI 0.17, 1.23). See Table 5.

**Table 5:** Multivariable model of predictors of Bayley Scale motor scores of 12 month old infants

	***Crude ***$\hat{\beta }$***(95%CI)*** *(n = 194)*	***Fully adjusted***$\hat{\beta }$***(95%CI) Model (n = 183)***
Maternal age (years)	-0.11 (-0.22,-0.0003)	-0.03 (-0.23, 0.18)
Age of husband (years)	-0.06 (-0.12, 0.002)	0.004 (-0.08, 0.09)
No maternal education	1.85 (-0.18, 3.88)	0.29 (-1.68, 2.26)
No paternal education	-0.70(-2.07, 0.67)	-0.35 (-1.83, 1.14)
Parity: Primiparous	Ref.^§^	Ref.^§^
2 -4 births	-1.13 (-3.51, 1.24)	-1.64 (-4.30, 1.01)
5 or more	-1.91 (-4.26, 0.43)	-2.03 (-5.53, 1.48)
Lower relative wealth	-2.10 (-3.46, -0.75)	-2.15 (-3.53, -0.77)
Hungry in last 30 days	-0.71 (-2.74, 1.32)	0.59 (-1.80, 2.97)
Marital discord scale	-0.65(-1.51, 0.21)	-0.17 (-1.26, 0.92)
Physically assaulted	-2.04 (-5.90, 1.82)	-1.62 (-7.24, 4.00)
Female infant gender	0.45 (-0.92, 1.82)	-0.39 (-1.87, 1.11)
Birth weight (kg)	-0.07(-1.68, 1.54)	-0.32 (-2.26, 1.63)
***Pregnancy variables***		
Maternal SRQ score	-0.20 (-0.37, -0.03)	-0.03 (-0.25, 0.19)
≥1 malarial episode	-0.15 (-2.55, 2.24)	0.55 (-1.93, 3.03)
***Postnatal 2 month variables***		
Maternal SRQ score	-0.23 (-0.52, 0.06)	-0.21, -0.50, 0.08)
Prolonged labour (>24 hours)	-1.47 (-3.27, 0.32)	-1.70 (-3.62, 0.22)
***Postnatal 12 month variables***		
Maternal SRQ score	-0.25 (-0.57, 0.06)	-0.13 (-0.46, 0.20)
Infant diarrhoea: 0	Ref.^±^	Ref.^±^
1 episode	1.17 (-0.79, 3.13)	1.37 (-0.72, 3.47)
≥2 episodes	0.37 (-1.29, 2.04)	0.83 (-1.07, 2.72)
Weight-for-Age Z-score	0.60 (0.14, 1.06)	0.70 (0.17, 1.23)

^§^Test-for-trend for parity: Χ^2 ^3.51 (1), p > 0.05 for crude and Χ^2 ^1.31(1), p > 0.25 for adjusted model.

^±^Test-for-trend for infant diarrhoeal episodes: Χ^2 ^0.004(1), p > 0.50 for crude and Χ^2 ^0.13(1), p > 0.50 for adjusted model.

## Discussion

This population-based prospective study from rural Ethiopia did not find evidence of an independent effect of maternal CMD in pregnancy or the postnatal period upon infant development at 12 months of age. There was also no evidence of effect modification by socio-economic status, infant gender or perceived social support. Moreover, the presence of high levels of CMD symptoms at more than one time-point ('persistent CMD') was not significantly associated with infant developmental outcomes after adjusting for confounding variables. Lower socio-economic status and infant weight-for-age were independently associated with poorer cognitive and motor development. Maternal experience of physical assault was associated only with poorer cognitive development.

### Study strengths and limitations

Given the low levels of health service contact by Ethiopian women of reproductive age, it was necessary that the study be population-based. The longitudinal design enabled consideration of direction of causality and reduced measurement bias. A particular strength of the study is that we were able to control for a range of important confounding variables and to distinguish effects of antenatal, postnatal, and persistent exposure to maternal CMD.

Some potential study limitations need to be borne in mind when interpreting our findings. First, the Western-derived measure of infant development, the Bayley Scales, may not have been culturally valid in this setting. Arguing against this as a major limitation are the previous reports of successful use of the Bayley Scales in Ethiopia [26,27]. Furthermore, a rigorous attempt to develop a more culturally valid measure of child development in another sub-Saharan African country, by adding new items and discarding those with poor test properties, found that the majority of items from the original Western scale could be adapted for use in the final scale [39]. Further support for the construct validity of the Bayley Scales comes from our finding that developmental scores were associated with expected predictors of developmental outcome. The lack of formal assessment of reliability of administration of the Bayley Scales was a further limitation, although a previous Ethiopian study demonstrated that careful training and monitoring of administrators, as was done in our study, may suffice [27]. Second, the assessment of language development may have been affected by the limited range of language typical of 12 month old infants [40] and by the necessity of relying on mothers' reports.

A third study limitation is the relatively small number of women with high levels of CMD symptoms in our sample, raising the possibility that we were under-powered to detect a true effect of maternal CMD on infant development. Fourth, overadjustment for confounding variables is a possible explanation for our negative finding. This could be a particular problem for variables that could be subject to recall bias, such as maternal and infant illness episodes. However, the lack of significant associations in the crude analyses argues against this as an important factor. Fifth, our use of maternal CMD symptoms as the measure of mental ill-health rather than restricting the exposure to depressive symptoms or a diagnosis of major depressive disorder might have diluted any effect on infant development [41]. However, previous studies relying on symptom scales have shown associations with impaired infant development [8,21,29,42]. Lastly, exploring features of the home environment and, specifically, characteristics of mother-infant interactions and the child-rearing practices of other caregivers would have added useful information on risk and protective factors that might modulate the effect of maternal CMD exposure upon infant development.

### Maternal CMD and child development

Our study findings are inconsistent with the results of a number of studies conducted in high-income countries [4,8,11,29,40,42] that have demonstrated adverse effects of maternal CMD, particularly in the postnatal period, upon infant and child cognitive and motor development. However, other well-conducted high-income country studies have either failed to replicate this association [5] or found that early exposure to maternal CMD explains little of the variability of the child developmental outcomes [29] or only detected an association in vulnerable sub-groups [12,16]. Furthermore, some studies only found an association between maternal CMD and specific cognitive tasks, for example, Piaget's object concept, and not with more global assessments of cognitive functioning [16,43].

Methodological differences may also go some way to explaining the lack of congruence with findings from previous LAMICs. Samples recruited from health facilities [19] might be biased towards more severe and chronic cases of maternal CMD. In the only other rural, prospective population-based study, based in Bangladesh [22], maternal CMD was not independently associated with infant development. The previous Ethiopian study was limited by the cross-sectional design and use of a non-validated measure of maternal CMD which may have led to measurement bias [21]. Differences in the timing of assessment of maternal CMD and infant development further complicate comparisons among studies. Later measurement of cognitive function may show the effects of maternal perinatal mental health more clearly [20].

Again contrary to some other studies from LAMICs [44], we have previously demonstrated a lack of association between maternal CMD and birthweight [23] and infant undernutrition [45] in this rural Ethiopian population. As infant undernutrition is an important risk factor for impaired development [1], this may explain the observed lack of association between maternal CMD and child development. Protective factors may prevent the negative consequences of risk factors upon infant development by influencing mediating factors; for example, mother-infant interaction, maternal cognition or characteristics of the home environment [46]. In this regard it is interesting to observe that available data on child-rearing practices in the sample population show that, although mothers are the primary care givers for the majority of infants, in a third of cases older siblings look after the infants for the longest time per day. Further exploration of potentially protective socio-cultural factors in the home environment and local community which may buffer negative effects of maternal mental health on infant development is indicated.

Although limited by the small numbers of women with persistent CMD in our study, there was some evidence to support an effect of chronicity of maternal CMD upon motor development. Univariate analyses found statistically significant associations, with little change in the size of the beta-coefficients when adjusting for a broad range of confounders; however, in the final adjusted model the confidence intervals were wide, rendering the result non-significant. The importance of distinguishing between mild and transient perinatal depressive symptoms and chronic mental disorders has been demonstrated in several studies, with effects on child development only seen with more enduring maternal mental disorder [8,29,40].

### Predictors of infant development

Our finding that perceived lower relative wealth is associated with poorer infant cognitive and motor development is in keeping with previous studies [15]. Similarly, poor infant nutritional status (stunting and under-weight) is an established risk factor for both cognitive and motor developmental delay [1]. This association has also been confirmed in three previous Ethiopian studies [21,26,27]. A growing body of evidence supports the importance of interpersonal violence during pregnancy for infant outcomes [47]. In our study, the effect of physical violence on infant cognitive outcome is unlikely to have been mediated through low birth weight as we were able to adjust for this in the multivariable analysis. A more probable explanation is continuity of violence from pregnancy into the postnatal period so that the developing child is exposed to the detrimental effects of ongoing family conflict [11].

### Research and policy implications

Our findings support the need to build capacity of primary health care workers providing ante- and postnatal services in LAMICs to identify mothers exposed to risk factors that may affect child development, particularly social adversity and violence. Psychosocial interventions to improve child development have already been successfully trialled in a sub-Saharan Africa setting [48]. The *P-MaMiE *cohort is continuing to be followed up with further assessments of child development in relation to maternal mental health, providing an opportunity to investigate the impact of chronicity of maternal CMD beyond the perinatal period. Exploration of potential protective factors that may contribute to infants' resilience to maternal CMD is also indicated.

## Conclusions

The study supports the hypothesis that it is the accumulation of risk exposures across time rather than early exposure to maternal CMD per se that is more likely to affect child development. In the Ethiopian setting, poverty, interpersonal violence and infant undernutrition should be targets for interventions to reduce the loss of child developmental potential.

## Competing interests

The authors declare that they have no competing interests.

## Authors' contributions

CH, MP, AA conceived the idea and designed the study; GM and CH co-ordinated data collection and data entry, with assistance from BW and YB; GM, CH, AA, BW, MT, MD and MP were involved in ongoing monitoring of the conduct of the study. GM, CH, MD, MP and CS analysed the data. CS and CH drafted the manuscript. GM, MD, BW, YB, MT, AA and MP critically commented on the draft manuscript. All authors contributed to the interpretation of results and approved the final manuscript

## Pre-publication history

The pre-publication history for this paper can be accessed here:

http://www.biomedcentral.com/1471-2458/10/693/prepub
